# Supplementary material for: miR-125a Promotes the Progression of Giant Cell Tumors of Bone by Stimulating IL-17A and β-Catenin Expression
Source: Mol Ther Nucleic Acids. 2018 Oct 2;13:493–502. doi: 10.1016/j.omtn.2018.09.021 (PMC6205328; doi:10.1016/j.omtn.2018.09.021)
Supplement: Document S1. Figure S1 and Tables S1–S5 [file mmc1.pdf]

OMTN, Volume *13*

Supplemental Information

miR-125a Promotes the Progression of Giant Cell Tumors of Bone by Stimulating IL-17A and β-Catenin Expression

Hua Jin, Dian-Wei Li, Shu-Nan Wang, Song Luo, Qing Li, Ping Huang, Jian-Min Wang, Meng Xu, and Cheng-Xiong Xu

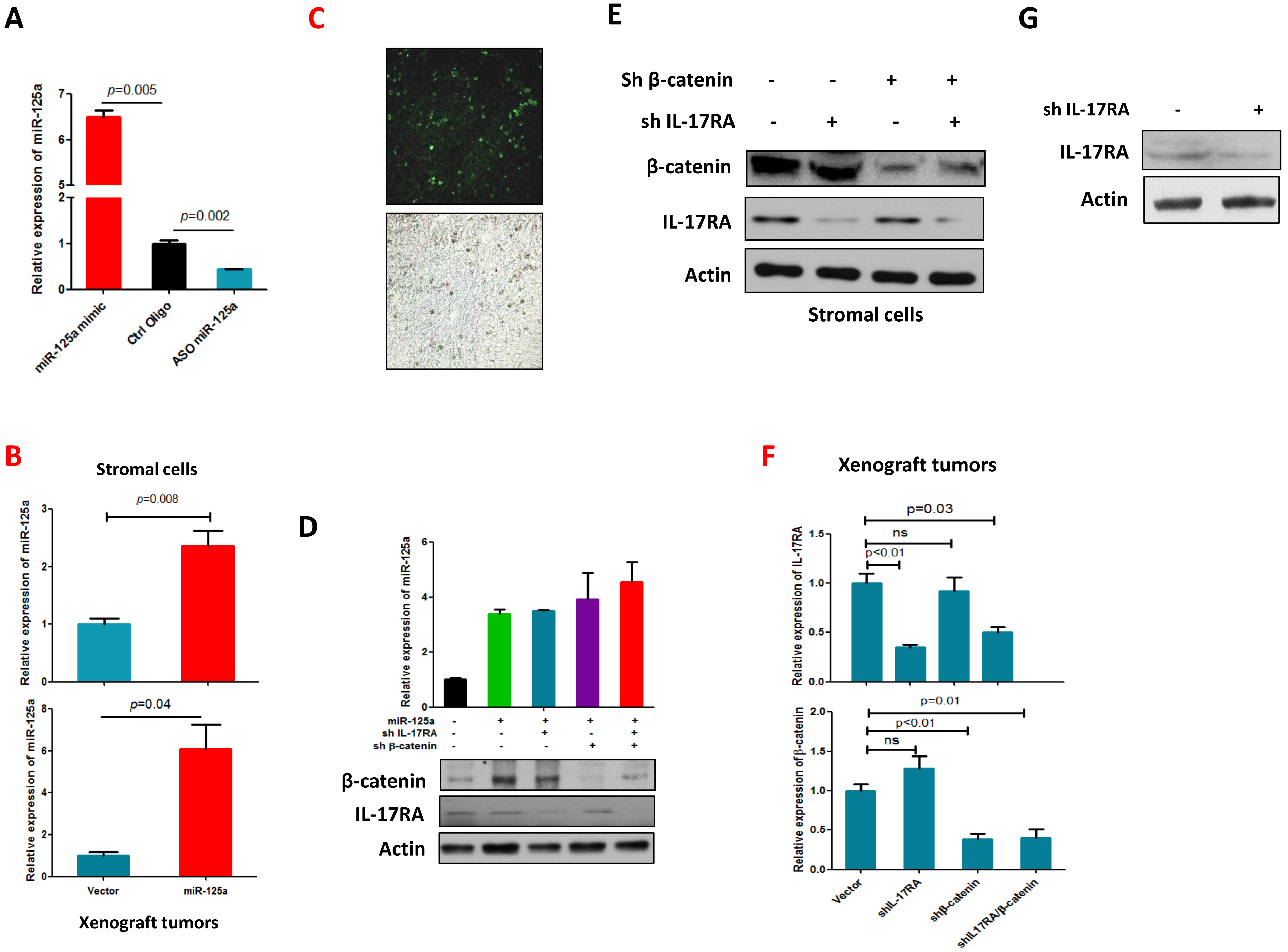

A
p=0.005
p=0.002
Relative expression of miR-125a
miR-125a mimic
Ctrl Oligo
ASO miR-125a
C
E
Sh β-catenin - - + +
sh IL-17RA - + - +
β-catenin
IL-17RA
Actin
Stromal cells
G
sh IL-17RA - +
IL-17RA
Actin
B
Stromal cells
p=0.008
p=0.04
Relative expression of miR-125a
Vector
miR-125a
Xenograft tumors
D
Relative expression of miR-125a
miR-125a - + + + +
sh IL-17RA - - + - +
sh β-catenin - - - + +
β-catenin
IL-17RA
Actin
F
Xenograft tumors
p=0.03
ns
p<0.01
Relative expression of IL-17RA
p=0.01
p<0.01
ns
Relative expression of β-catenin
Vector
shIL-17RA
shβ-catenin
shIL17RA/β-catenin

Supplementary Table 1. The association of miR-125a expression with clinical parameters

Variable	miR-125a		
	High	Low	*p*
Sex			0.425
Male	34	31	
Female	26	16	
Age			0.416
≤30	37	33	
>30	23	14	
Pathologic fracture			0.820
Yes	14	10	
No	46	37	
Tumor extension			0.014
T1(Intracompartmental)	33	37	
T2(Extracompartmental)	27	10	
Campanacci grade			0.018
Grade I/II	31	35	
Grade III	29	12	

X^2 test

Supplementary Table 2. The risk factor of recurrence

Variable	Univariate analysis		Multivariate analysis	
	HR (95% CI)	*p* value	HR (95% CI)	*p* value
Age	1.44 (0.74-2.82)	0.283	1.06（0.51-2.17）	0.882
Gender	0.69 (0.34-1.41)	0.306	0.57（0.27-1.22）	0.148
Campanacci Frade	1.22 (0.98-1.52)	0.084	1.15（0.91-1. 44）	0.253
Tumor extersion	1.72 (0.89-3.35)	0.109	1.36（0.67-2.78）	0.396
Pathologic fracture	1. 01 (0.46 -2.22)	0.982	1.36（0.56-3.32）	0.500
IL-17A	3.69 (1.53-8.89)	0.004	2.78（1.02-7.53）	0.045
MiR-125	4.65 (1.93-11.21)	0.001	2.96（1.12-7.84）	0.029

Supplementary Table 3. Demographics and clinical variables

Characteristic	N（%）
Sex	
Male	65（60.7）
Female	42（39.3）
Age	
≤30	70（65.4）
>30	37（34.6）
Pathologic fracture	
Yes	24（22.4）
No	83（77.6）
Tumor extension	
T1(Intracompartmental)	70（65.4）
T2(Extracompartmental)	37（34.6）
Campanacci grade	
Grade I//II	66（61.7）
Grade III	41（38.3）
Recurrence	
Yes	35（32.7）
No	72（67.3）

Supplementary Table 4. qRT-PCR primer sequences that used in this study.

Genes	Forward	Reverse
IL-17A	AACCGATCCACCTCACCTTG	TCTCTTGCTGGATGGGGACA
APC	GGAAGCAGAGAAAGTACTGGA	CTGAAGTTGAGCGTAATACCAG
GSK3β	GACTAAGGTCTTCCGACCCC	TTAGCATCTGACGCTGCTGT
TET2	TGTTGTTGTCAGGGTGAGAATC	TCTTGCTTCTGGCAAACTTACA
Foxp3	TGCAGGGCAGCTAGGTACTTG	TCGGAGATCCCCTTTGTCTTATC
IL-17RA	ATGGACACTGCAGACAGACG	CTCACAGTCAGGCACAAGGA
β-catenin	ACAAACTGTTTTGAAAATCCA	CGAGTCATTGCATACTGTCC
GAPDH	ATTCCATGGCACCGTCAAGGCTG	TTCTCCATGGTGGTGAAGACGCCA

Supplementary Table 5. PCR primer sequences for target genes 3-UTR cloning

Genes	Prime sequences
APC	AAAGATCCTTTATTAAGCTTAAGAGAGGAAGAATGAAACTAAG
	GCGCACTAGTGAGGGAGCTCTTTTTATTACAGTAAGAGGAATTTTACTC
GSK3β	AAAGATCCTTTATTAAGCTTTTGTTTTCTTTTAAAGTCTAGTGTGAGACTTTG
	GCGCACTAGTGAGGGAGCTCCAAAGAAAACAGACACAAAAACTCTTAAC
TET2	AAAGATCCTTTATTAAGCTTAACAGGGTTAGTTCCATGTGAATCTG
	GCGCACTAGTGAGGGAGCTCTAAATACTAGTAAGCAAAAATGTATTTAGGCCAAAATG
FOXP3	AAAGATCCTTTATTAAGCTTCCTCAAGATCAAGGAAAGGAGGATG
	GCGCACTAGTGAGGGAGCTCTGTGGGGAGCTCGGCTGCAG

SUPPLEMENTARY FIGURE LEGENDS

Supplementary Figure 1. Transfection efficiency. (**A)** The expression level of miR-125a was measured in stromal cells using qRT-PCR after 72 hours of transfection with miR-125a mimics or antisense nucleotides of miR-125a (ASO miR-125a). (**B)** The expression level of miR-125a was measured in stromal cells and xenograft tumors. Stromal cells were infected with or without miR-125a expression lentiviral vector. After 72 hours of infection, cells were subjected to qRT-PCR analysis. Tumors were collected from xenograft tumors that generated by miR-125a-overexpressing stromal cells, then subjected to qRT-PCR analysis (Support Figs. 2D and E). **(C)** The green fluorescence expressed in the tumor tissues was examined using a fluorescence microscope. Xenograft tumors were generated using stromal cells that transfected with miR-125a sponge-expressing vector (contain EGFP sequences) and the tissues were collected after 2 months of cell injection.**(D)** Stromal cells were infected with indicated lentiviral vectors. After 72 hours of infection, cells were subjected to qRT-PCR and Western blot analysis. (**E)** Stromal cells were infected with IL-17RA or/and β-catenin shRNA expression lentiviral vectors. After 72 hours of infection, cells were subjected to Western blot analysis. (**F**) The mRNA expressions of IL-17RA and β-catenin were measured in xenograft tumors by qRT-PCR analysis. Tumors were collected from xenograft models generated by miR-125a-overexpressing stromal cells that infected with shRNA of IL-17RA or/and β-catenin (Support Fig. 3F). (**G)** Stromal cells were transfected with IL-17RA shRNA expression lentiviral vector or scramble vector. After 72 hours of infection, cells were subjected to Western blot analysis.
