# Supplementary material for: miR-125a Promotes the Progression of Giant Cell Tumors of Bone by Stimulating IL-17A and β-Catenin Expression
Source: Mol Ther Nucleic Acids. 2018 Oct 2;13:493–502. doi: 10.1016/j.omtn.2018.09.021 (PMC6205328; doi:10.1016/j.omtn.2018.09.021)
Supplement: Document S2. Article plus Supplemental Information [file mmc2.pdf]

Molecular Therapy
Nucleic Acids
Original Article

miR-125a Promotes the Progression of Giant Cell Tumors of Bone by Stimulating IL-17A and β-Catenin Expression

Hua Jin,[1,7] Dian-Wei Li,[2,7] Shu-Nan Wang,[3] Song Luo,[4] Qing Li,[5] Ping Huang,[5] Jian-Min Wang,[6] Meng Xu,[4] and Cheng-Xiong Xu[5]

[1]Department of Thoracic Surgery, Daping Hospital and Research Institute of Surgery, Third Military Medical University, Chongqing 400042, China; [2]Department of Orthopaedics, The SouthWest Hospital, Third Military Medical University, Chongqing 400038, China; [3]Department of Radiology, Daping Hospital and Research Institute of Surgery, Third Military Medical University, Chongqing 400042, China; [4]Department of Orthopaedics, The General Hospital of Chinese People's Liberation Army, Beijing 100853, China; [5]Cancer Center, Daping Hospital and Research Institute of Surgery, Third Military Medical University, Chongqing 400042, China; [6]State Key Laboratory of Trauma, Burn and Combined Injury, Daping Hospital and Research Institute of Surgery, Third Military Medical University, Chongqing 400042, China

Giant cell tumors of bone (GCTBs) exhibit high recurrence and aggressive bone lytic behavior; but, the mechanism of GCTB progression is largely unknown. In GCTB, we detected abundant levels of miR-125a, which were associated with tumor extension, grade, and recurrence. miR-125a stimulates stromal cell tumorigenicity and growth *in vivo* by promoting the expression of interleukin-17A (IL-17A) and β-catenin. In contrast, inhibition of miR-125a suppressed stromal cell tumorigenicity and growth. Then, we found that miR-125a stimulates IL-17A by targeting TET2 and Foxp3, and it stimulates β-catenin expression by targeting APC and GSK3β in stromal cells. Furthermore, we identified that IL-17A stimulates miR-125a by activating nuclear factor κB (NF-κB) signaling in stromal cells. Finally, our data show that simultaneous inhibition of IL-17A signaling and miR-125a more significantly inhibits stromal cell growth than miR-125a inhibition alone. miR-125a stimulates the progression of GCTB, and it might represent a useful candidate marker for progression. Simultaneously blocking miR-125a and IL-17A might represent a new therapeutic strategy for GCTB.

INTRODUCTION

Giant cell tumor of bone (GCTB) is a benign, locally aggressive, osteolytic tumor that causes significant bone destruction in the epiphysis of long bones. The major challenge in the clinical treatment of GCTB is recurrence. The primary method for GCTB treatment in the clinic is surgery. However, according to one report, 20%–50% of patients with GCTB exhibit local recurrence following primary surgical treatments, up to 5% of patients develop pulmonary metastases, and 2% develop spontaneous malignant transformation.[1] Unfortunately, there are no specific methods or drugs that can effectively inhibit the recurrence of GCTB, and no known biomarker can predict its progression. Additionally, the cellular mechanism that leads to GCTB development and progression remains unclear.

GCTB consists of three major cell types: osteoclast-like multinucleated giant cells (MNGCs), monocytic round-shaped macrophage-like cells, and spindle-shaped fibroblast-like stromal cells.[2] The bone resorption activity of GCTB is primarily attributed to the MNGCs, the development and activity of which are significantly regulated by stromal cells via a complex network of cytokines and chemokines.[1,2] These findings suggest that both MNGCs and stromal cells play important roles in GCTB development and progression. Thus, both MNGCs and stromal cells are important therapeutic targets in GCTB treatment.

Recent studies showed that aberrantly increased expression of interleukin-17A (IL-17A) and β-catenin are closely associated with GCTB progression, including tumor growth, bone destruction, and recurrence.[3,4] However, the regulatory mechanism of IL-17A and β-catenin in GCTB is not fully understood. MicroRNAs (miRNAs) comprise a class of small non-coding RNAs that negatively regulate the expression of target genes at the post-transcriptional level through interaction with the 3′ UTRs of the target genes.[5] Dysregulated expression of miRNAs has been detected in most cancers, and certain aberrantly expressed miRNAs play key roles in cancer initiation and progression. Dysregulated miRNAs are also detected in GCTB,[6] and several miRNAs have been implicated in the progression of GCTB.[7] However, unlike other tumors, the role of miRNAs in GCTB is largely unknown. Interestingly, although not previously demonstrated in GCTBs, certain dysregulated miRNAs are involved in the regulation

Received 21 March 2018; accepted 26 September 2018;
https://doi.org/10.1016/j.omtn.2018.09.021.

[7]These authors contributed equally to this work.

Correspondence: Cheng-Xiong Xu, Cancer Center, Daping Hospital and Research Institute of Surgery, Third Military Medical University, 10 Changjiang Zhilu, Daping, Yuzhong District, Chongqing 400042, China.
E-mail: xuchengxiong@hanmail.net
Correspondence: Meng Xu, Department of Orthopaedics, The General Hospital of Chinese People's Liberation Army, 28 Fuxing Road, Beijing 100853, China.
E-mail: xumeng301@gmail.com

This is an open access article under the CC BY license (http://creativecommons.org/licenses/by/4.0/).

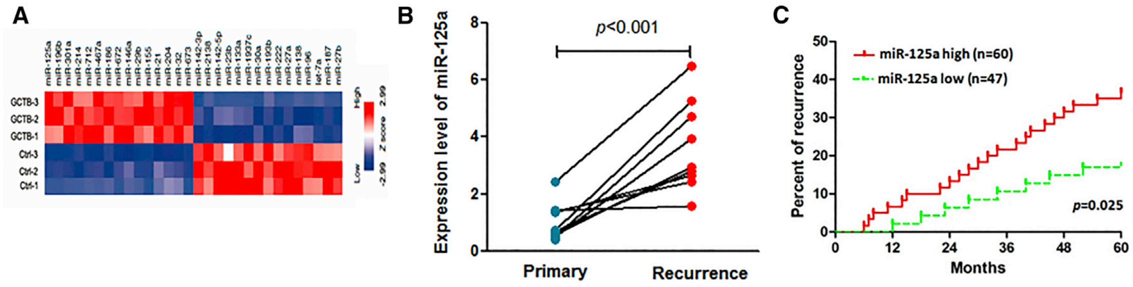

Figure 1. Increased Expression Level of miR-125a Is Significantly Correlated with GCTB Recurrence

(A) miRNA expression heatmap. An miRNA array was performed using recurrent GCTB specimens and corresponding normal bone tissues (n = 3). (B) Using qRT-PCR to measure miR-125a expression in recurrent GCTB specimens and corresponding primary GCTB specimens (n = 10). (C) The correlation between miR-125a level and the recurrence rate in 107 patients with GCTB was analyzed using the Kaplan-Meier method.

of IL-17A and β-catenin expression and functions.[8–10] Based on these findings, we speculated that dysregulated miRNAs may be involved in the regulation of IL-17A and β-catenin expression and function in GCTBs, thereby stimulating GCTB progression. Thus, in this study, we demonstrated that miRNAs play a key role in the progression of GCTB and are involved in the regulation of IL-17A and β-catenin expression.

RESULTS

Aberrantly Increased Expression of miR-125a in GCTB Was Correlated with Disease Progression

To identify GCTB progression-associated miRNAs, we performed miRNAs array analysis using recurrent GCTB tissues and their matched normal bone tissues from 3 patients. As shown in Figure 1A, miR-125a was significantly increased in GCTB tissues compared to their adjacent normal tissues. In addition, our data show that the miR-125a expression level was significantly higher in recurrent GCTB tissues compared to the primary tumor tissues (Figure 1B). Consistent with these results, we identified a higher recurrence rate in the miR-125a high-expressing GCTB patient group compared to the miR-125a low-expressing patient group (Figure 1C). Additionally, we found that miR-125a was more frequently overexpressed in aggressive and extracompartmental extension lesions (Table S1). Furthermore, the univariate and multivariate analyses showed that miR-125a level was significantly associated with GCTB recurrence (Table S2). These findings suggested that increased miR-125a might be involved in the stimulation of GCTB progression, including recurrence.

miR-125a Significantly Stimulates GCTB Progression

The stromal cells are the only proliferating population in GCTBs, and the expression of miR-125a was significantly increased in stromal cells compared to normal bone (Figure 2A). Thus, to investigate whether miR-125a was directly involved in GCTB progression, we overexpressed or inhibited miR-125a in primary cultured stromal cells (Figure S1A), and then we performed a cell viability assay. Our data showed that overexpression of miR-125a significantly promoted stromal cell growth, while inhibition of miR-125a had the opposite effect (Figure 2B). Consistent with this finding, a soft agar assay also showed that overexpression or inhibition of miR-125a increased or inhibited stromal cell colony formation ability, respectively (Figure 2C). Furthermore, we confirmed these results observed *in vitro* using animal experiments. To investigate the effects of miR-125a on tumor growth and tumorigenicity *in vivo*, two doses of miR-125a-overexpressing stromal cells (Figure S1B), miR-125a sponge-transfected stromal cells (Figure S1C), and their corresponding vector control cells were subcutaneously inoculated into nude mice. As shown in Figures 2D and 2E, miR-125a-overexpressing stromal cells displayed higher tumorigenicity and formed larger tumors than the vector control stromal cells. In contrast, miR-125a-inhibited stromal cells showed weakened tumorigenicity and formed smaller tumors than the vector control stromal cells (Figures 2F and 2G). These results strongly supported the notion that miR-125a is a tumor promotion miRNA that significantly contributes to GCTB progression.

miR-125a Stimulates GCTB Progression by Increasing the Expression of β-Catenin and IL-17A in Stromal Cells

Previous studies have shown that β-catenin and IL-17A are risk factors for GCTB progression.[3,4] Thus, we investigated whether the expression level of miR-125a correlated with the expression of β-catenin and IL-17A in GCTB. As shown in Figure 3A, overexpression of miR-125a increased while inhibition of miR-125a suppressed β-catenin expression in stromal cells. Similar to β-catenin, we detected a high or low concentration of IL-17A in the conditioned media from the miR-125a-overexpressing or miR-125a-inhibited stromal cells compared to control stromal cells, respectively (Figure 3B). Additionally, we identified a positive correlation between the expression level of β-catenin (Figure 3C) and IL-17A (Figure 3D) with miR-125a in human GCTB specimens. These data suggest that miR-125a positively regulates β-catenin and IL-17A expression in GCTB.

Next, to investigate whether β-catenin and IL-17A directly contribute to miR-125a function in GCTB, we silenced β-catenin and IL-17 receptor A (IL-17RA) in miR-125a-overexpressing stromal cells (Figure S1D), and then we performed a cell viability assay. As shown in Figure 3E, miR-125a-stimulated stromal cell growth was significantly inhibited by silencing of β-catenin and/or IL-17RA, indicating that miR-125a-induced stromal cell growth stimulation is dependent on β-catenin and IL-17A. Furthermore, we confirmed these *in vitro*

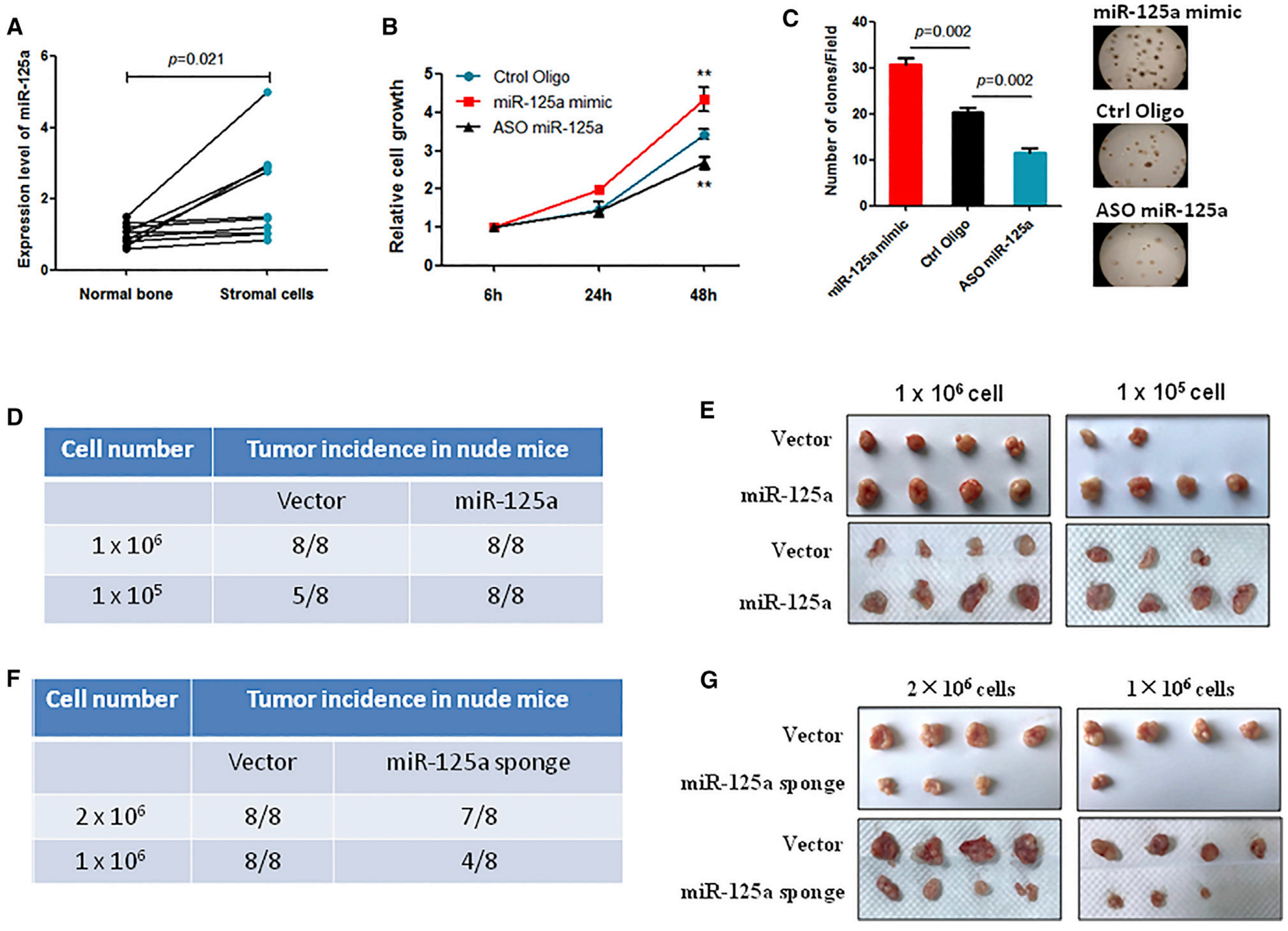

Figure 2. miR-125a Stimulates Stromal Cell Tumorigenicity and Tumor Growth

(A) miR-125a expression was measured by qRT-PCR in normal bone tissues and stromal cells obtained from ten GCTB patient specimens. (B) A cell viability assay was performed using stromal cells after overexpression or inhibition of miR-125a. (C) A soft agar assay was performed using stromal cells after overexpression or inhibition of miR-125a. (D) Tumor incidence in nude mice injected with stromal cells that were transfected with an miR-125a expression lentiviral vector. Mice were sacrificed 2 months after cell injection. (E) Representative images of tumors are shown. (F) Tumor incidence in nude mice injected with stromal cells that were transfected with an miR-125a sponge expression lentiviral vector. Mice were sacrificed 2 months after cell injection. (G) Representative images of tumors are shown.

experimental results *in vivo* using an miR-125a-overexpressing stromal cell xenograft model. Consistent with the *in vitro* results, β-catenin- or IL-17RA-silenced (Figures S1E and S1F) miR-125a-overexpressing stromal cells showed weakened tumorigenicity and formed smaller tumors than control cells (Figure 3F). Notably, simultaneous silencing of β-catenin and IL-17RA more strongly inhibited tumorigenicity and tumor growth compared to single-gene silencing in miR-125a-overexpressing stromal cell xenografts (Figure 3F). These data clearly indicate that miR-125a-stimulated tumor growth and tumorigenicity are mediated through increasing β-catenin and IL-17A expression in GCTB.

miR-125a Targeting Negative Regulators of β-Catenin and IL-17A in Stromal Cells

To investigate how miR-125a enhances IL-17A and β-catenin expression, we screened miR-125a target genes using computational algorithm analysis, and we identified Foxp3, TET2, APC, and GSK3β as potential target genes of miR-125a (Figure 4A). APC and GSK3β are well studied negative regulators of β-catenin, and recent studies showed that Foxp3 inhibits IL-17A expression[11] and TET2 stimulates Foxp3 expression.[12] In addition, previous studies showed that TET2[13] and Foxp3[14] are targets of miR-125a, although not in GCTB. However, no studies have shown whether TET2 and Foxp3 are expressed in GCTB. Thus, we first investigated whether TET2 and Foxp3 are expressed in GCTB specimens. Our immunohistochemistry (IHC) results clearly showed that TET2 was expressed in both MNGCs and stromal cells, while Foxp3 was only expressed in stromal cells (Figure 4B), suggesting that TET2 and Foxp3 may be involved in the regulation of IL-17A expression in stromal cells.

To confirm the negative regulation of miR-125a on these candidate target genes, we measured the expression level of the candidate target

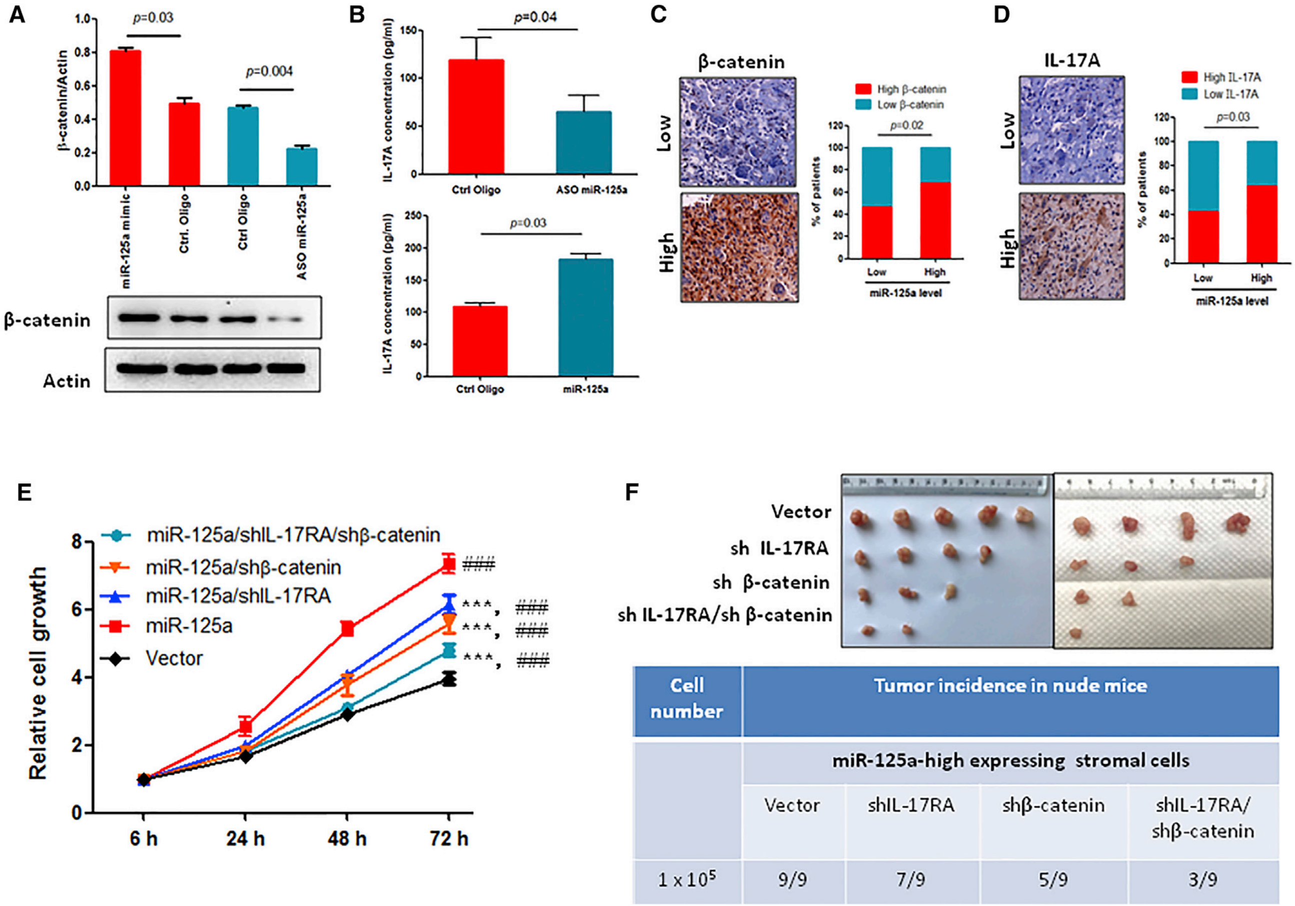

Figure 3. The Expression Level of miR-125a Is Correlated with IL-17A and β-Catenin Levels in GCTB

(A) The expression of β-catenin was measured using western blotting in stromal cells 72 hr after transfection with the indicated oligonucleotides. (B) The IL-17A concentration in the stromal cell cultured media was measured. The stromal cells were transfected with miR-125a mimic or antisense miR-125a, and the media were changed 48 hr later. After an additional 48 hr, the IL-17A concentration was measured. (C) The expression level of β-catenin was measured in miR-125a high-expression specimens (n = 60) and low-expression specimens (n = 47) by IHC. (D) The expression level of IL-17A was measured in miR-125a high-expression specimens (n = 60) and low-expression specimens (n = 47) by IHC. (E) The stromal cells were infected with the indicated lentiviral vectors, and then we performed cell viability assays at the indicated times. ###p < 0.001 (compared to vector control), ***p < 0.001 (compared to miR-125a group). (F) Tumor incidence in nude mice injected with miR-125a high-expressing stromal cells that were infected with the indicated gene shRNA expression lentiviral vector. Mice were sacrificed 2 months after cell injection.

genes after overexpression of miR-125a in stromal cells. Our data showed that all candidate genes of miR-125a were significantly decreased in miR-125a-overexpressing stromal cells compared to control at both the mRNA and protein levels (Figures 4C and 4D). Then, we confirmed the direct binding between miR-125a and the 3′ UTR of candidate target genes by luciferase assay. Each 3′ UTR of the candidate target genes, harboring the complementary sequence to the miR-125a seed sequence, was cloned into a reporter plasmid. Transient cotransfection of each candidate target gene-3′ UTR construct with miR-125a into stromal cells led to a significant decrease in firefly luciferase activity compared to control. However, mutation of the putative target site in the 3′ UTR abolished this repression by miR-125a (Figure 4E), indicating that miR-125a negatively regulates the expression of APC, GSK3β, TET2, and Foxp3 by directly targeting the 3′ UTR of target genes in stromal cells.

Next, we investigated whether these target genes directly contribute to miR-125a-induced expression and function of IL-17A and β-catenin. Our data showed that overexpression of TET2 or Foxp3 significantly abolished the miR-125a-induced IL-17A expression in stromal cells (Figure 4F). Notably, co-overexpression of TET2 and Foxp3 more significantly inhibited the miR-125a-induced IL-17A expression level compared to single-gene overexpression (Figure 4F). Additionally, TET2 and/or Foxp3 overexpression abolished IL-17A-induced receptor activator of nuclear factor κB ligand (RANKL) expression while inhibiting IL-17A-induced inhibition of osteoprotegerin (OPG) expression in stromal cells (Figure 4F). Additionally, overexpression of the miR-125a target gene APC and/or GSK3β significantly abolished miR-125a overexpression-induced β-catenin expression (Figure 4G). These findings suggest that miR-125a stimulates IL-17A expression by targeting TET2 and Foxp3 and

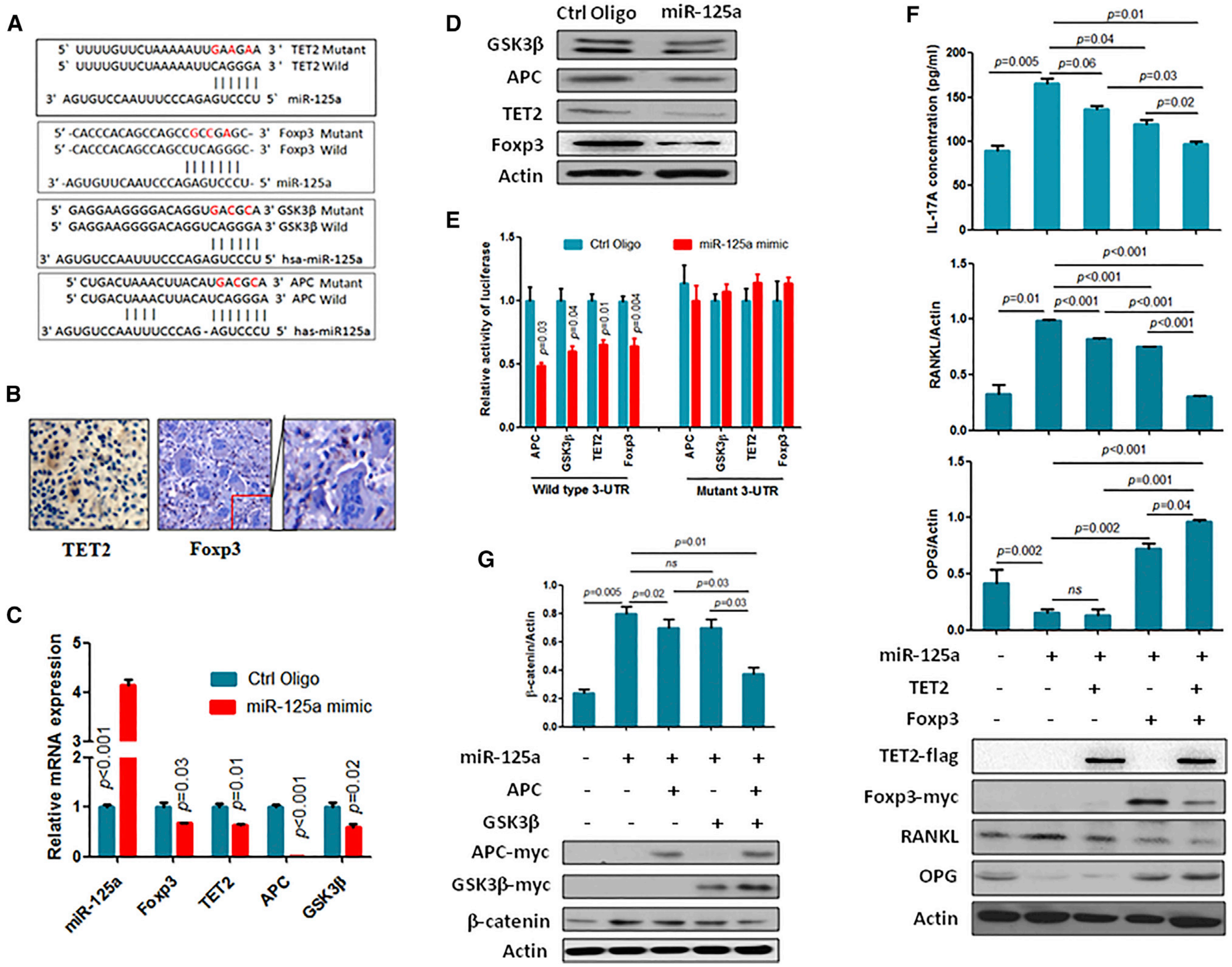

Figure 4. miR-125a Directly Targets Multiple Negative Regulators of IL-17A and β-Catenin

(A) Predicted binding sites of miR-125a in the wild-type 3′ UTRs of Foxp3, TET2, GSK3β, and APC. Mutations in these 3′ UTRs are highlighted in red. (B) TET2 and Foxp3 expression was detected in GCTB specimens by IHC. (C) After 72 hr of transfection with miR125a mimics, the stromal cells were subjected to qRT-PCR analysis for detecting the indicated gene's mRNA level. (D) After 72 hr of transfection with miR125a mimics, the stromal cells were subjected to western blot analysis to detect the indicated protein's level. (E) Luciferase activity of reporter with wild-type or mutant 3′ UTRs of Foxp3, TET2, APC, and GSK3β in the stromal cells cotransfected with the indicated oligonucleotides. (F) Stromal cells were infected with the indicated gene expression lentiviral vectors. After 72 hr of infection, cell culture medium and cells were subjected to measurement of IL-17A concentration and western blot analysis, respectively. (G) After 72 hr of infection with the indicated gene expression lentiviral vectors, the stromal cells were subjected to western blot analysis for detecting the indicated protein's expression.

stimulates β-catenin expression by targeting APC and GSK3β in stromal cells.

IL-17A Stimulates miR-125a Expression by the Activation of NF-κB Signaling

Although not conducted in GCTB, previous studies showed that miR-17A activates nuclear factor κB (NF-κB) signaling, and activated NF-κB signaling is involved in the regulation of miRNAs, including miR-125a.[15,16] Thus, here we investigated whether IL-17A is involved in the regulation of miR-125a expression in GCTB. As shown in Figure 5A, IL-17A treatment dose-dependently increased miR-125a expression in stromal cells. In addition, silencing of IL-17RA (Figure S1G) significantly abolished IL-17A-induced miR-125a expression in stromal cells (Figure 5B), indicating that miR-125a expression was partly regulated by IL-17A in stromal cells. Then, we investigated whether IL-17A stimulates miR-125a expression through NF-κB signaling. Consistent with findings in other tumors, our data showed that miR-17A significantly decreased cytoplasmic p65 while increasing nuclear p65 in stromal cells (Figure 5C). In addition, activation of NF-κB signaling by tumor necrosis factor alpha (TNF-α) stimulated the expression of miR-125a, and the TNF-α-stimulated miR-125a expression was abolished by NF-κB inhibitor

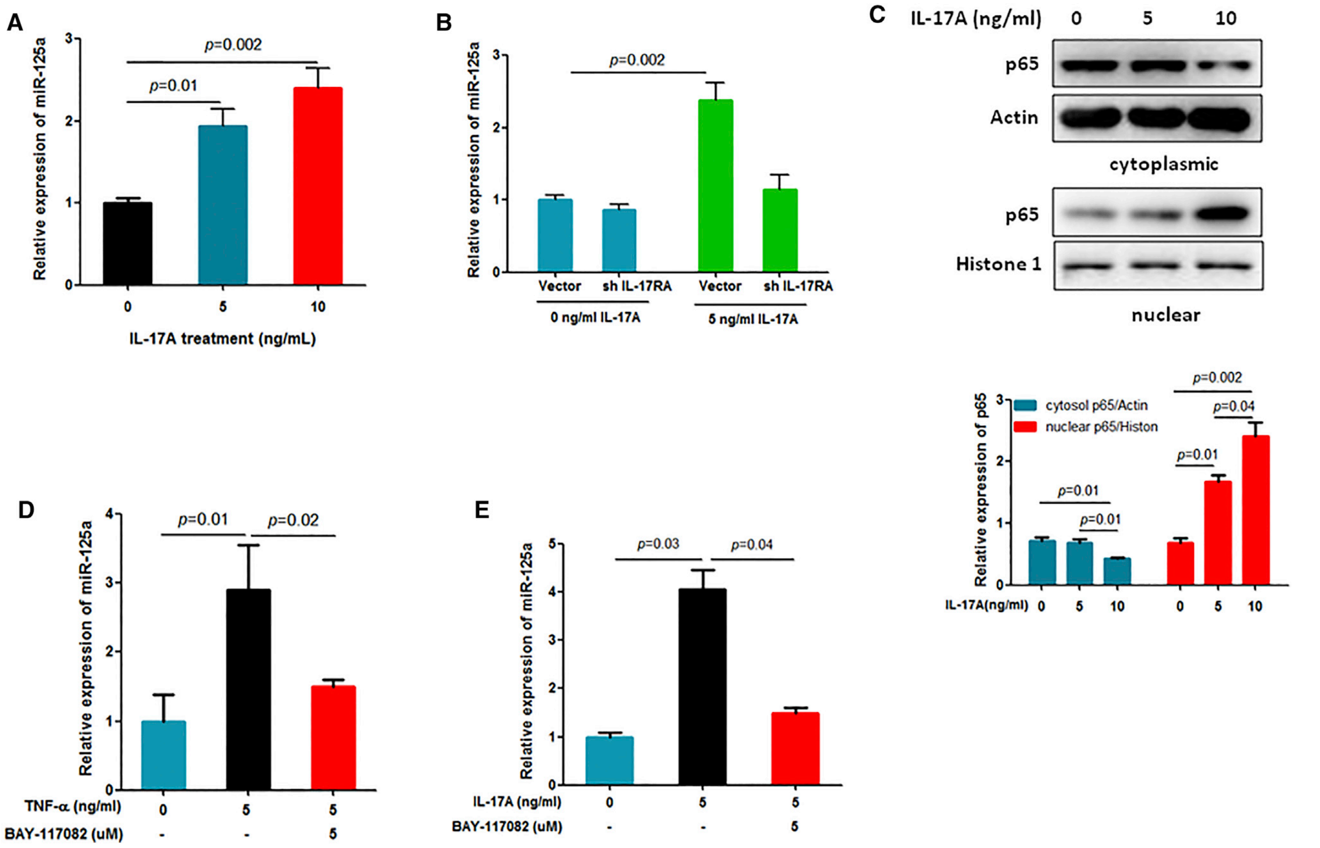

Figure 5. IL-17A Stimulates miR-125a Expression in Stromal Cells by Activating NF-κB Signaling

(A) Stromal cells were treated with the indicated concentration of IL-17A for 24 hr, and then the cells were subjected to qRT-PCR analysis to detect the miR-125a expression level. (B) Stromal cells were transfected with the scramble vector or IL-17RA shRNA expressing lentiviral vector, treated with 5 ng/mL IL-17A for 24 hr, and subjected to qRT-PCR to detect the miR-125a expression level. (C) Stromal cells were treated with the indicated concentration of IL-17A for 24 hr, and then we isolated cytoplasmic and nuclear proteins to detect the p65 protein level by western blot analysis. (D) The stromal cells were treated with 5 ng/mL TNF-α with or without 5 μM BAY-117082 for 6 hr. Total RNA was harvested, and miR-125a expression was measured by real-time RT-PCR. (E) Stromal cells were treated with 5 ng/mL IL-17A with or without 5 μM BAY-117082 for 6 hr. Total RNA was harvested, and miR-125a expression was measured by real-time RT-PCR.

BAY-117082 treatment of stromal cells (Figure 5D). Furthermore, our data showed that IL-17A-induced expression of miR-125a was also abolished by BAY-117082 treatment in stromal cells (Figure 5E). These results clearly indicate that IL-17A stimulates miR-125a expression by activating NF-κB signaling in stromal cells.

Simultaneously Blocking IL-17A and miR-125a Significantly Inhibits GCTB Progression

Our previous study showed that IL-17A is expressed in both stromal cells and MNGCs and IL-17A can stimulate its self-expression in MNGCs.[3] In addition, although not previously studied in GCTB, IL-17A can stimulate β-catenin expression.[17,18] Thus, here we investigated whether IL-17A affects IL-17A and β-catenin expression in stromal cells. Our data showed that IL-17A significantly stimulated the expression of IL-17A (Figure 6A) and β-catenin in stromal cells (Figure 6B). These data suggest that simultaneously blocking miR-125a and IL-17A may inhibit GCTB progression better than blocking miR-125a alone. As expected, simultaneously blocking miR-125a and IL-17RA most effectively inhibited miR-125a high-expressing stromal cell viability compared to single-gene inhibition in the presence of IL-17A (Figure 6C).

DISCUSSION

In this study, we found that the expression level of miR-125a was significantly increased in GCTB specimens compared to normal bone tissues. In addition, we showed that an increased expression level of miR-125a was closely correlated with GCTB grade, extension, and recurrence in patients. These findings suggest miR-125a is a tumor-promoting miRNA and can be a prognostic marker for GCTB progression.

Both MNGCs and stromal cells play important roles in GCTB progression. Notably, recent studies have suggested that stromal cells may be involved in GCTB development and recurrence. According to these reports, stromal cells are the only proliferating population in GCTB, and they express osteoblast factors,[19,20] contain subpopulations with stem cell-like properties,[21,22] and can form tumors in nude mice.[3] In

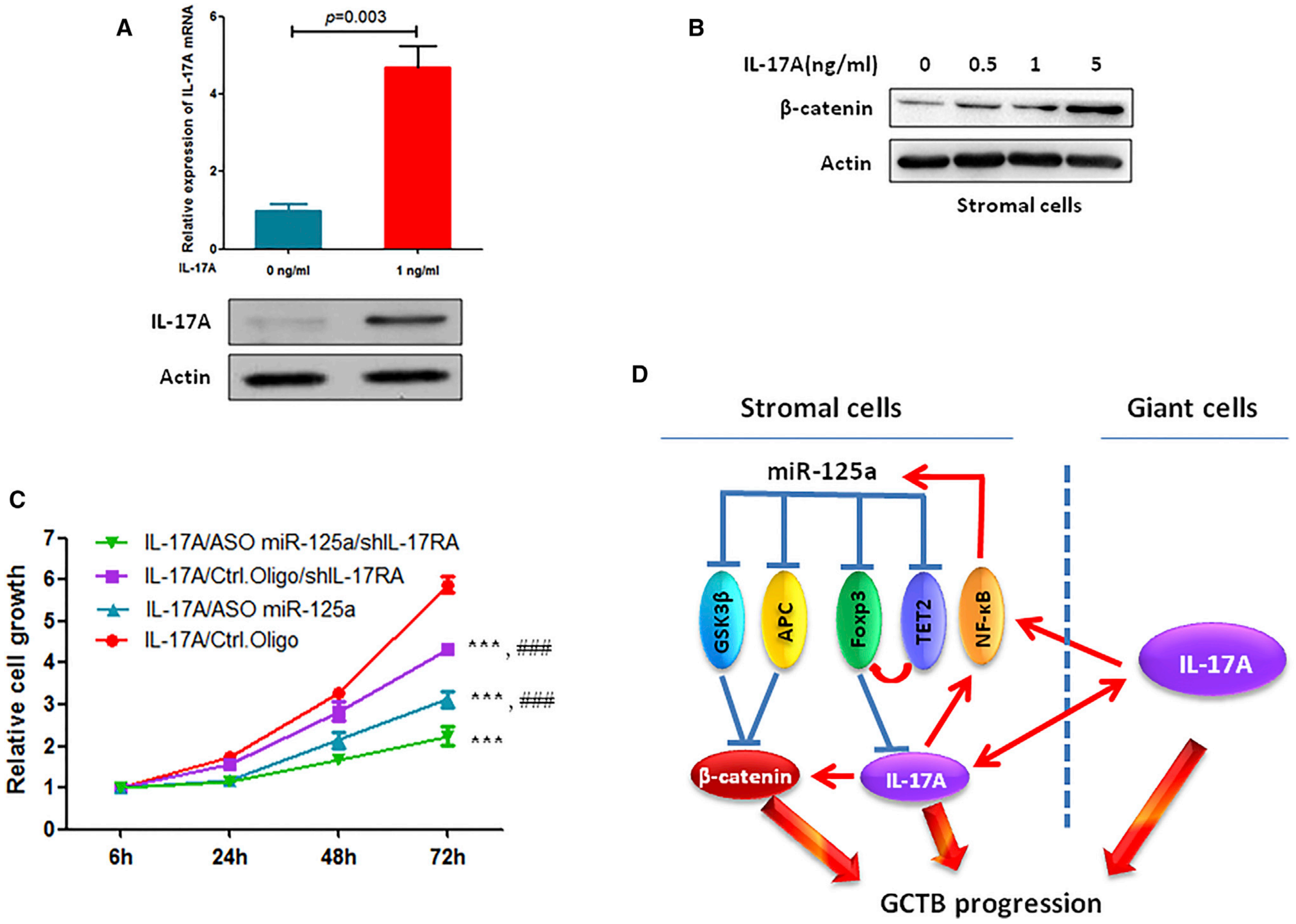

Figure 6. Simultaneously Blocking IL-17A and miR-125a More Strongly Inhibited Stromal Cell Growth

(A) IL-17A stimulates IL-17A expression in stromal cells. Stromal cells were treated with 1 ng/mL IL-17A for 24 hr, and the IL-17A expression in stromal cells was measured using qRT-PCR and western blot. (B) IL-17A stimulates β-catenin expression in stromal cells. Stromal cells were treated with the indicated concentration of IL-17A for 24 hr and then subjected to western blot analysis. (C) Stromal cells were transfected with the indicated nucleotides and/or lentiviral vectors and treated with 1 ng/mL IL-17A, and then cell viability was measured at the indicated times. Ctrl. Oligo, control oligonucleotides; ###p < 0.001 (compared to IL-17A/ASO miR-125a/shIL-17RA group); ***p < 0.001 (compared to IL-17A/Ctrl. Oligo group). (D) A schematic model of the regulation of GCTB progression by miR-125a and IL-17A.

recent years, new treatment options have been developed, such as the monoclonal antibody denosumab directed against RANKL; denosumab does not affect the GCTB recurrence rate because it mainly affects the bone destructive osteoclast-like giant cells within the tumor, rather than the stromal cells.[7,23,24] Here we used a series of *in vitro* and *in vivo* experiments to demonstrate that aberrantly increased expression of miR-125a stimulates stromal cell growth and tumorigenicity. Importantly, the inhibition of miR-125a significantly inhibited stromal cell tumorigenicity and tumor growth in a xenograft model. Taken together, our findings indicate that miR-125a is a novel therapeutic target of stromal cells and inhibition of miR-125a is a novel strategy to suppress GCTB progression.

GCTB is characterized by a high recurrence rate, and studies have shown that aberrantly highly expressed β-catenin and IL-17A are closely associated with GCTB progression, including tumor growth and recurrence.[3,4] Thus, understanding the biological basis for the deregulation of β-catenin and IL-17A is of great value for the future development of novel therapeutic strategies. Here we demonstrated that overexpression of miR-125a substantially increased the expression of β-catenin and IL-17A in stromal cells and promoted stromal cell growth and tumorigenicity through β-catenin and IL-17A. In addition, we showed that miR-125a stimulates β-catenin expression, by targeting the β-catenin negative regulators APC and GSK3β, and IL-17A expression, by targeting TET2 and Foxp3 in stromal cells. Taken together, miR-125a stimulates GCTB progression by increasing β-catenin and IL-17A expression through targeting their multiple inhibitors, providing a new layer of molecular mechanisms by which miR-125a promotes GCTB progression.

In a previous study, we demonstrated that IL-17A was secreted by both stromal cells and MNGCs, thereby significantly promoting

GCTB progression.[3] In this study, we detected Foxp3 expression in stromal cells, but not in MNGCs, indicating that IL-17A expression in MNGCs is not dependent on miR-125a/Foxp3 signaling. Additionally, these results suggest that miR-125a inhibition cannot suppress the expression of IL-17A by MNGCs. Notably, our data show that IL-17A significantly stimulates miR-125a expression through activating NF-κB signaling. Additionally, IL-17A stimulates IL-17A and β-catenin expression in stromal cells, suggesting that simultaneous targeting of miR-125a and IL-17A is more effective in GCTB treatment. In fact, our results showed that simultaneously blocking miR-125a and IL-17RA more strongly inhibited stromal cell growth in medium containing added IL-17A compared to the miR-125a inhibition group.

In summary, our study, to the best of our knowledge, is the first to have delineated the correlation between miR-125a and the progression of GCTBs. miR-125a stimulates the progression of GCTBs through increasing the expression of β-catenin and IL-17A by directly targeting their negative regulators. Our results suggest that miR-125a is a useful progression marker of GCTB and simultaneously blocking miR-125a and IL-17A might represent a new therapeutic strategy for GCTB.

MATERIALS AND METHODS

Patient Samples and the Evaluation of GCTB Progression in Patients

Tumor samples were obtained from 107 patients with GCTB, and all patients received extended curettage, with no adjuvant therapy. The clinical characteristics of the patients with GCTB are summarized in Table S3. The progression of the GCTB was evaluated using the Campanacci grading[25] and Enneking staging systems.[26] This research was approved by the Research Ethics Board of the General Hospital of the People's Liberation Army, China.

Primary Stromal Cell Isolation and Culture

The stromal cells were isolated from fresh tumor tissues of GCTB patients as described previously,[3] and they were cultured in DMEM containing 4.5 g/L glucose with 10% fetal calf serum and 100 U/mL penicillin-streptomycin (Sigma, St. Louis, MO, USA). Each experiment was performed using stromal cells that were from 3 GCTB patients.

Cell Viability and Soft Agar Assay

Stromal cells were transfected with the indicated nucleotides (Ribo Bio, Guangzhou, China) or lentiviral vectors. After 24 hr of transfection, the cells were trypsinized. For the cell viability assay, the cells were reseeded in 96-well plates at a density of 5,000/well, and then the cell viability was measured at the indicated times using Cell Counting Kit-8 (MedChem Express, NJ, USA), according to the manufacturer's instructions.

For the soft agar assay, cells were re-suspended in 0.5 mL 0.35% agar (Sigma) in growth medium at a density of 5,000 cells/well (6-well plates). Then, the agar-cell mixture was plated on the top of a solid layer of 0.8% agar in growth medium. Colonies were counted 14 days later.

Microarray and Real-Time qPCR Analysis

RNA was isolated from cells and human specimens using the TRIzol reagent (Invitrogen, Carlsbad, CA, USA) or miRNeasy Mini Kits (QIAGEN, Hilden, Germany), according to the manufacturer's protocol. Microarray analysis was performed using LC MicroRNA microarray Chip_H11.0_081344, which detects miRNA transcripts listed in Sanger miRBase Release 11.0. Imaging and quantifying analysis were performed using an Affymetrix scanner and a fag software.

For miR-125a and RNU6B, reverse transcription and qPCR were performed with The TaqMan MicroRNA Reverse Transcription Kit and TaqMan Universal PCR Master Mix, respectively, using Ambion miRNA primers. The relative expression of miR-125a was normalized against RNU6 expression using the $2^{-\Delta Ct}$ method, and the miR-125a expression fold change in GCTB samples matched to nontumor control samples was evaluated using the $2^{-\Delta\Delta Ct}$ method. Based on the mean fold change of miR-125a expression, patients were divided into high (fold change > mean) and low (fold change < mean) miR-125a expression groups.[27] For other gene expression, reverse transcription and qPCR were performed with a High Capacity cDNA Reverse Transcription Kit and QuantiTect SYBR Green PCR kit, respectively. All materials for qRT-PCR were purchased from Life Technologies (Carlsbad, CA, USA). The primer sequences for the genes are provided in Table S4.

Constructs

3′ UTR segments of miR-125a target genes that were predicted to interact with miR-125a were amplified by PCR from human genomic DNA and inserted into the Hind III and Sac I sites of the miRNA Expression Reporter Vector (Life Technologies). The primer sequences are provided in Table S5.

Pre-miR-125a-encoding sequences were subcloned into the GV838 lentiviral vector (GENECHEM, Shanghai, China) and lentiviral vectors. For the miR-125a sponge, synthetic oligonucleotides containing five tandem repeats of bulged miR-125a-binding sites were ligated into the GV369 lentiviral vector (contain EGFP sequences) (GENECHEM). The sequence of the control sponge was as follows: 5′-ACTAGTTAATGTTAGCTAAGAGAAAACTTCGATTGTTAG CTAAGAGAAAACTTCGATTGTTAGCTAAGAGAAAACTTCGAT TGTTAGCTAAGAGAAAACTTCGATTGTTAGCTAAGAGAAA ACTTCGATTGTTAGCTAAGAGAAAACTTCGCGGATCC-3′. APC and GSK3β expression constructs were purchased from GeneCopoeia (Rockville, MD, USA). Small hairpin RNA (shRNA) of β-catenin and IL-17RA lentiviral vectors were obtained from Santa Cruz Biotechnology (Dallas, TX, USA). The TET2 construct was generated as described by Ito et al.[28]

Luciferase Reporter Assay

For the luciferase assay, stromal cells were cotransfected with the indicated reporter plasmids and either the miR-125a mimics or control

oligonucleotides. These cells were also cotransfected with a renilla luciferase plasmid as an internal control. Luciferase activity was measured 72 hr post-transfection using a Dual-Luciferase Assay Kit (Promega, Madison, WI, USA), according to the manufacturer's protocol, and the luciferase activity was normalized to the activity of renillar luciferase.

Western Blot and IHC

Western blot analysis and IHC were conducted as previously described.[29] Total, cytoplasmic, and nuclear proteins were extracted using protein extraction kits (101Bio, Palo Alto, CA, USA), and all antibodies were purchased from Abcam (Cambridge, MA, USA). Western blot bands were quantified using ImageJ software.

IL-17A Assay

The IL-17A in the media of primary cultured cells was measured using a human IL-17A ELISA kit (Abcam), according to the manufacturer's instructions.

Animal Models

The stromal cells were infected with the indicated lentiviral vectors. After 48 hr of infection, cells were injected subcutaneously into 6-week-old female nude mice. At 2 months after the cell injection, the mice were sacrificed.

Statistical Analysis

Kaplan-Meier survival analysis and x^2 tests were used for calculating the recurrence rate of GCTB and the association of miR-125a expression with clinical parameters, respectively. All of the other results are expressed as the mean values ± SD. Statistical significance was analyzed by unpaired Student's t tests or one-way ANOVA and Duncan's multiple range tests using SAS statistical software package version 6.12 (SAS Institute); $p \leq 0.05$ was considered to be statistically significant.

SUPPLEMENTAL INFORMATION

Supplemental Information includes one figure and five tables and can be found with this article online at https://doi.org/10.1016/j.omtn.2018.09.021.

AUTHOR CONTRIBUTIONS

H.J., M.X., and C.-X.X. contributed to the conception and design of the study. S.L., D.-W.L., Q.L., and M.X., collected the human samples and performed IHC. H.J., D.-W.L., and P.H. performed laboratory and animal experiments. H.J., S.-N.W., J.-M.W., and C.-X.X. analyzed the data. H.J. and C.-X.X. wrote the manuscript.

CONFLICTS OF INTEREST

The authors have no conflicts of interest.

ACKNOWLEDGMENTS

This work was supported by the National Natural Science Foundation of China (81402216 to M.X. and 81672657 to C.-X.X.), Beijing Newstar Plan of Science and Technology (Z14110700181409 to M.X.), the Military Youth Innovation Training Program (16QNP145 to M.X.), the Translational Medicine Program of GHPLA (2016TM-019 to M.X.), the National Defense Program (3408080 to M.X.), and the Startup Fund for Talented Scholars of Daping Hospital and Research Institute of Surgery, Third Military Medical University (to C.-X.X. and H.J.).

REFERENCES

1. Turcotte, R.E. (2006). Giant cell tumor of bone. Orthop. Clin. North Am. *37*, 35–51.
2. Werner, M. (2006). Giant cell tumour of bone: morphological, biological and histogenetical aspects. Int. Orthop. *30*, 484–489.
3. Xu, M., Song, Z.G., Xu, C.X., Rong, G.H., Fan, K.X., Chen, J.Y., Zhang, W., Jia, J.P., Han, G., Wang, W., et al. (2013). IL-17A stimulates the progression of giant cell tumors of bone. Clin. Cancer Res. *19*, 4697–4705.
4. Matsubayashi, S., Nakashima, M., Kumagai, K., Egashira, M., Naruke, Y., Kondo, H., Hayashi, T., and Shindo, H. (2009). Immunohistochemical analyses of beta-catenin and cyclin D1 expression in giant cell tumor of bone (GCTB): a possible role of Wnt pathway in GCTB tumorigenesis. Pathol. Res. Pract. *205*, 626–633.
5. Garzon, R., Calin, G.A., and Croce, C.M. (2009). MicroRNAs in Cancer. Annu. Rev. Med. *60*, 167–179.
6. Mosakhani, N., Pazzaglia, L., Benassi, M.S., Borze, I., Quattrini, I., Picci, P., and Knuutila, S. (2013). MicroRNA expression profiles in metastatic and non-metastatic giant cell tumor of bone. Histol. Histopathol. *28*, 671–678.
7. Fellenberg, J., Sähr, H., Kunz, P., Zhao, Z., Liu, L., Tichy, D., and Herr, I. (2016). Restoration of miR-127-3p and miR-376a-3p counteracts the neoplastic phenotype of giant cell tumor of bone derived stromal cells by targeting COA1, GLE1 and PDIA6. Cancer Lett. *371*, 134–141.
8. Du, C., Liu, C., Kang, J., Zhao, G., Ye, Z., Huang, S., Li, Z., Wu, Z., and Pei, G. (2009). MicroRNA miR-326 regulates TH-17 differentiation and is associated with the pathogenesis of multiple sclerosis. Nat. Immunol. *10*, 1252–1259.
9. Matsuyama, H., Suzuki, H.I., Nishimori, H., Noguchi, M., Yao, T., Komatsu, N., Mano, H., Sugimoto, K., and Miyazono, K. (2011). miR-135b mediates NPM-ALK-driven oncogenicity and renders IL-17-producing immunophenotype to anaplastic large cell lymphoma. Blood *118*, 6881–6892.
10. Jin, H., Luo, S., Wang, Y., Liu, C., Piao, Z., Xu, M., Guan, W., Li, Q., Zou, H., Tan, Q.Y., et al. (2017). miR-135b Stimulates Osteosarcoma Recurrence and Lung Metastasis via Notch and Wnt/β-Catenin Signaling. Mol. Ther. Nucleic Acids *8*, 111–122.
11. Liao, J., Liu, Y., Wu, H., Zhao, M., Tan, Y., Li, D., Long, H., Dai, Y., Yung, S., Chan, T.M., and Lu, Q. (2016). The role of icaritin in regulating Foxp3/IL17a balance in systemic lupus erythematosus and its effects on the treatment of MRL/lpr mice. Clin. Immunol. *162*, 74–83.
12. Yue, X., Trifari, S., Äijö, T., Tsagaratou, A., Pastor, W.A., Zepeda-Martínez, J.A., Lio, C.W., Li, X., Huang, Y., Vijayanand, P., et al. (2016). Control of Foxp3 stability through modulation of TET activity. J. Exp. Med. *213*, 377–397.
13. Cheng, J., Guo, S., Chen, S., Mastriano, S.J., Liu, C., D'Alessio, A.C., Hysolli, E., Guo, Y., Yao, H., Megyola, C.M., et al. (2013). An extensive network of TET2-targeting MicroRNAs regulates malignant hematopoiesis. Cell Rep. *5*, 471–481.
14. Li, J., Qiu, D., Chen, Z., Du, W., Liu, J., and Mo, X. (2016). Altered expression of miR-125a-5p in thymoma-associated myasthenia gravis and its down-regulation of foxp3 expression in Jurkat cells. Immunol. Lett. *172*, 47–55.
15. Potenza, N., Panella, M., Castiello, F., Mosca, N., Amendola, E., and Russo, A. (2017). Molecular mechanisms governing microRNA-125a expression in human hepatocellular carcinoma cells. Sci. Rep. *7*, 10712.
16. Ghose, J., and Bhattacharyya, N.P. (2015). Transcriptional regulation of microRNA-100, -146a, and -150 genes by p53 and NFκB p65/RelA in mouse striatal STHdh(Q7)/Hdh(Q7) cells and human cervical carcinoma HeLa cells. RNA Biol. *12*, 457–477.
17. Hyun, Y.S., Han, D.S., Lee, A.R., Eun, C.S., Youn, J., and Kim, H.Y. (2012). Role of IL-17A in the development of colitis-associated cancer. Carcinogenesis *33*, 931–936.

18. Zhang, Q., Liu, S., Parajuli, K.R., Zhang, W., Zhang, K., Mo, Z., Liu, J., Chen, Z., Yang, S., Wang, A.R., et al. (2017). Interleukin-17 promotes prostate cancer via MMP7-induced epithelial-to-mesenchymal transition. Oncogene *36*, 687–699.

19. Huang, L., Teng, X.Y., Cheng, Y.Y., Lee, K.M., and Kumta, S.M. (2004). Expression of preosteoblast markers and Cbfa-1 and Osterix gene transcripts in stromal tumour cells of giant cell tumour of bone. Bone *34*, 393–401.

20. Ghert, M., Simunovic, N., Cowan, R.W., Colterjohn, N., and Singh, G. (2007). Properties of the stromal cell in giant cell tumor of bone. Clin. Orthop. Relat. Res. *459*, 8–13.

21. Lan, J., Liu, X., Rong, W., Wei, F., Jiang, L., Yu, H., Dang, G., and Liu, Z. (2012). Stro-1(+) stromal cells have stem-like features in giant cell tumor of bone. J. Surg. Oncol. *106*, 826–836.

22. Liu, L., Aleksandrowicz, E., Fan, P., Schönsiegel, F., Zhang, Y., Sähr, H., Gladkich, J., Mattern, J., Depeweg, D., Lehner, B., et al. (2014). Enrichment of c-Met+ tumorigenic stromal cells of giant cell tumor of bone and targeting by cabozantinib. Cell Death Dis. *5*, e1471.

23. Thomas, D., Henshaw, R., Skubitz, K., Chawla, S., Staddon, A., Blay, J.Y., Roudier, M., Smith, J., Ye, Z., Sohn, W., et al. (2010). Denosumab in patients with giant-cell tumour of bone: an open-label, phase 2 study. Lancet Oncol. *11*, 275–280.

24. Traub, F., Singh, J., Dickson, B.C., Leung, S., Mohankumar, R., Blackstein, M.E., Razak, A.R., Griffin, A.M., Ferguson, P.C., and Wunder, J.S. (2016). Efficacy of denosumab in joint preservation for patients with giant cell tumour of the bone. Eur. J. Cancer *59*, 1–12.

25. Campanacci, M., Baldini, N., Boriani, S., and Sudanese, A. (1987). Giant-cell tumor of bone. J. Bone Joint Surg. Am. *69*, 106–114.

26. Enneking, W.F., Spanier, S.S., and Goodman, M.A. (2003). A system for the surgical staging of musculoskeletal sarcoma. 1980. Clin. Orthop. Relat. Res. (415), 4–18.

27. Li, J., Huang, H., Sun, L., Yang, M., Pan, C., Chen, W., Wu, D., Lin, Z., Zeng, C., Yao, Y., et al. (2009). MiR-21 indicates poor prognosis in tongue squamous cell carcinomas as an apoptosis inhibitor. Clin. Cancer Res. *15*, 3998–4008.

28. Ito, S., D'Alessio, A.C., Taranova, O.V., Hong, K., Sowers, L.C., and Zhang, Y. (2010). Role of Tet proteins in 5mC to 5hmC conversion, ES-cell self-renewal and inner cell mass specification. Nature *466*, 1129–1133.

29. Xu, C.X., Jere, D., Jin, H., Chang, S.H., Chung, Y.S., Shin, J.Y., Kim, J.E., Park, S.J., Lee, Y.H., Chae, C.H., et al. (2008). Poly(ester amine)-mediated, aerosol-delivered Akt1 small interfering RNA suppresses lung tumorigenesis. Am. J. Respir. Crit. Care Med. *178*, 60–73.

OMTN, Volume *13*

Supplemental Information

miR-125a Promotes the Progression of Giant Cell Tumors of Bone by Stimulating IL-17A and β-Catenin Expression

Hua Jin, Dian-Wei Li, Shu-Nan Wang, Song Luo, Qing Li, Ping Huang, Jian-Min Wang, Meng Xu, and Cheng-Xiong Xu

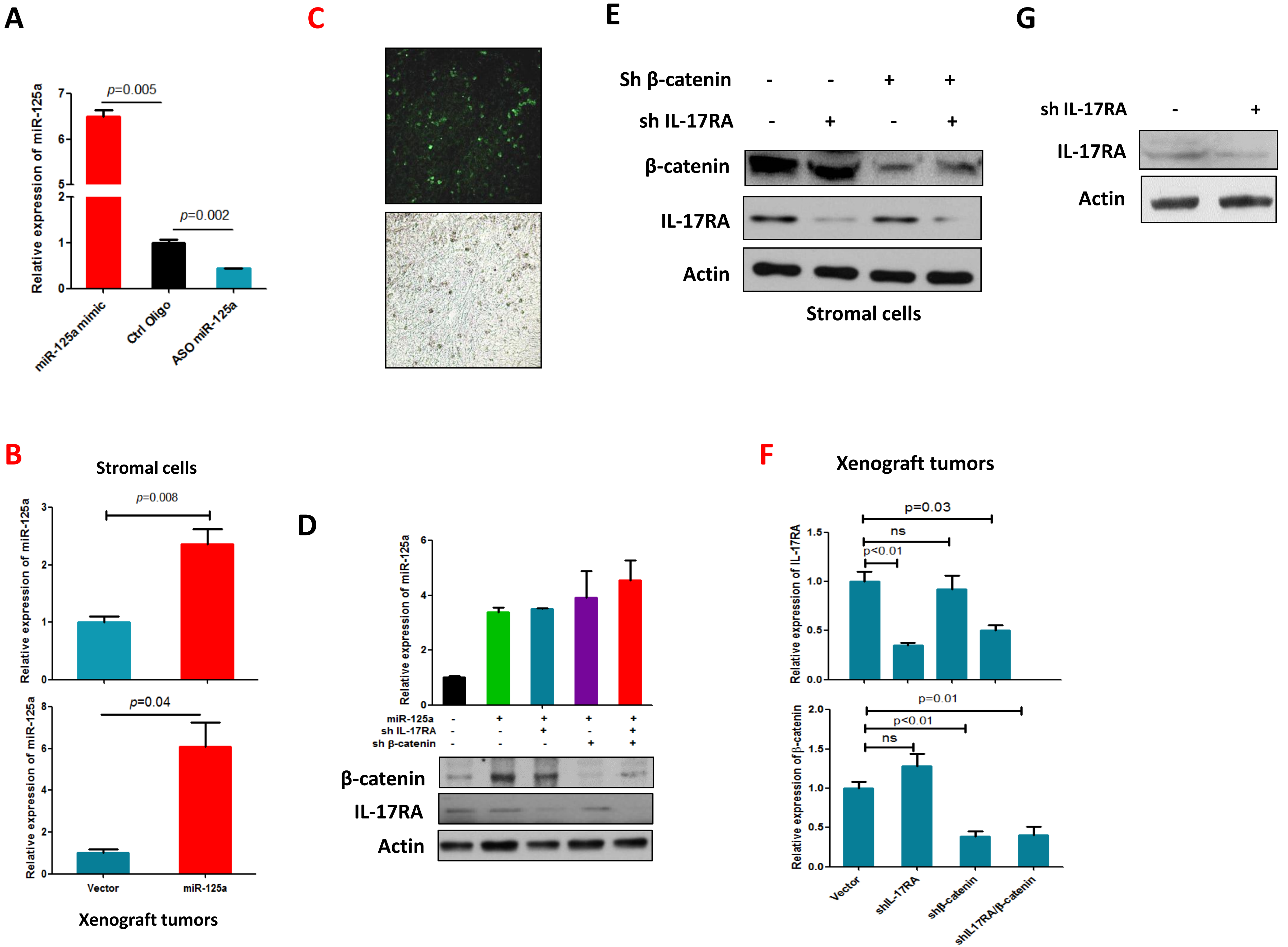
A
Relative expression of miR-125a
p=0.005
p=0.002
miR-125a mimic
Ctrl Oligo
ASO miR-125a
C
E
Sh β-catenin - - + +
sh IL-17RA - + - +
β-catenin
IL-17RA
Actin
Stromal cells
G
sh IL-17RA - +
IL-17RA
Actin
B
Stromal cells
p=0.008
p=0.04
Vector
miR-125a
Xenograft tumors
D
Relative expression of miR-125a
miR-125a - + + + +
sh IL-17RA - - + - +
sh β-catenin - - - + +
β-catenin
IL-17RA
Actin
F
Xenograft tumors
p=0.03
ns
p<0.01
Relative expression of IL-17RA
p=0.01
p<0.01
ns
Relative expression of β-catenin
Vector
shIL-17RA
shβ-catenin
shIL17RA/β-catenin

Supplementary Table 1. The association of miR-125a expression with clinical parameters

Variable	miR-125a High	miR-125a Low	*p*
Sex			0.425
Male	34	31	
Female	26	16	
Age			0.416
≤30	37	33	
>30	23	14	
Pathologic fracture			0.820
Yes	14	10	
No	46	37	
Tumor extension			0.014
T1(Intracompartmental)	33	37	
T2(Extracompartmental)	27	10	
Campanacci grade			0.018
Grade I/II	31	35	
Grade III	29	12	

X^2 test

Supplementary Table 2. The risk factor of recurrence

Variable	Univariate analysis		Multivariate analysis	
	HR (95% CI)	*p* value	HR (95% CI)	*p* value
Age	1.44 (0.74-2.82)	0.283	1.06（0.51-2.17）	0.882
Gender	0.69 (0.34-1.41)	0.306	0.57（0.27-1.22）	0.148
Campanacci Frade	1.22 (0.98-1.52)	0.084	1.15（0.91-1. 44）	0.253
Tumor extersion	1.72 (0.89-3.35)	0.109	1.36（0.67-2.78）	0.396
Pathologic fracture	1. 01 (0.46 -2.22)	0.982	1.36（0.56-3.32）	0.500
IL-17A	3.69 (1.53-8.89)	0.004	2.78（1.02-7.53）	0.045
MiR-125	4.65 (1.93-11.21)	0.001	2.96（1.12-7.84）	0.029

Supplementary Table 3. Demographics and clinical variables

Characteristic	N（%）
Sex	
Male	65（60.7）
Female	42（39.3）
Age	
≤30	70（65.4）
>30	37（34.6）
Pathologic fracture	
Yes	24（22.4）
No	83（77.6）
Tumor extension	
T1(Intracompartmental)	70（65.4）
T2(Extracompartmental)	37（34.6）
Campanacci grade	
Grade I//II	66（61.7）
Grade III	41（38.3）
Recurrence	
Yes	35（32.7）
No	72（67.3）

Supplementary Table 4. qRT-PCR primer sequences that used in this study.

Genes	Forward	Reverse
IL-17A	AACCGATCCACCTCACCTTG	TCTCTTGCTGGATGGGGACA
APC	GGAAGCAGAGAAAGTACTGGA	CTGAAGTTGAGCGTAATACCAG
GSK3β	GACTAAGGTCTTCCGACCCC	TTAGCATCTGACGCTGCTGT
TET2	TGTTGTTGTCAGGGTGAGAATC	TCTTGCTTCTGGCAAACTTACA
Foxp3	TGCAGGGCAGCTAGGTACTTG	TCGGAGATCCCCTTTGTCTTATC
IL-17RA	ATGGACACTGCAGACAGACG	CTCACAGTCAGGCACAAGGA
β-catenin	ACAAACTGTTTTGAAAATCCA	CGAGTCATTGCATACTGTCC
GAPDH	ATTCCATGGCACCGTCAAGGCTG	TTCTCCATGGTGGTGAAGACGCCA

Supplementary Table 5. PCR primer sequences for target genes 3-UTR cloning

Genes	Prime sequences
APC	AAAGATCCTTTATTAAGCTTAAGAGAGGAAGAATGAAACTAAG
	GCGCACTAGTGAGGGAGCTCTTTTTATTACAGTAAGAGGAATTTTACTC
GSK3β	AAAGATCCTTTATTAAGCTTTTGTTTTCTTTTAAAGTCTAGTGTGAGACTTTG
	GCGCACTAGTGAGGGAGCTCCAAAGAAAACAGACACAAAAACTCTTAAC
TET2	AAAGATCCTTTATTAAGCTTAACAGGGTTAGTTCCATGTGAATCTG
	GCGCACTAGTGAGGGAGCTCTAAATACTAGTAAGCAAAAATGTATTTAGGCCAAAATG
FOXP3	AAAGATCCTTTATTAAGCTTCCTCAAGATCAAGGAAAGGAGGATG
	GCGCACTAGTGAGGGAGCTCTGTGGGGAGCTCGGCTGCAG

SUPPLEMENTARY FIGURE LEGENDS

Supplementary Figure 1. Transfection efficiency. (**A**) The expression level of miR-125a was measured in stromal cells using qRT-PCR after 72 hours of transfection with miR-125a mimics or antisense nucleotides of miR-125a (ASO miR-125a). (**B**) The expression level of miR-125a was measured in stromal cells and xenograft tumors. Stromal cells were infected with or without miR-125a expression lentiviral vector. After 72 hours of infection, cells were subjected to qRT-PCR analysis. Tumors were collected from xenograft tumors that generated by miR-125a-overexpressing stromal cells, then subjected to qRT-PCR analysis (Support Figs. 2D and E). **(C)** The green fluorescence expressed in the tumor tissues was examined using a fluorescence microscope. Xenograft tumors were generated using stromal cells that transfected with miR-125a sponge-expressing vector (contain EGFP sequences) and the tissues were collected after 2 months of cell injection.**(D)** Stromal cells were infected with indicated lentiviral vectors. After 72 hours of infection, cells were subjected to qRT-PCR and Western blot analysis. (**E**) Stromal cells were infected with IL-17RA or/and β-catenin shRNA expression lentiviral vectors. After 72 hours of infection, cells were subjected to Western blot analysis. (**F**) The mRNA expressions of IL-17RA and β-catenin were measured in xenograft tumors by qRT-PCR analysis. Tumors were collected from xenograft models generated by miR-125a-overexpressing stromal cells that infected with shRNA of IL-17RA or/and β-catenin (Support Fig. 3F). (**G**) Stromal cells were transfected with IL-17RA shRNA expression lentiviral vector or scramble vector. After 72 hours of infection, cells were subjected to Western blot analysis.
